# *De novo* annotation of the transcriptome of the Northern Wheatear (*Oenanthe oenanthe*)

**DOI:** 10.7717/peerj.5860

**Published:** 2018-11-20

**Authors:** Roberto Carlos Frias-Soler, Lilian Villarín Pildaín, Agnes Hotz-Wagenblatt, Jonas Kolibius, Franz Bairlein, Michael Wink

**Affiliations:** 1 Institute of Pharmacy and Molecular Biotechnology, Heidelberg University, Heidelberg, Baden Württemberg, Germany; 2 Institute of Avian Research, Wilhelmshaven, Germany; 3 Bioinformatics Group, Core Facility Genomics and Proteomics, German Cancer Research Center, Heidelberg University, Heidelberg, Baden Württemberg, Germany

**Keywords:** *Oenanthe oenanthe*, Migratory birds, RNASeq, Transcriptome, Northern wheatears, Migratory phenotype, Migratory syndrome

## Abstract

We have sequenced a partial transcriptome of the Northern Wheatear (*Oenanthe oenanthe*), a species with one of the longest migrations on Earth. The transcriptome was constructed *de novo* using RNA-Seq sequence data from the pooled mRNA of six different tissues: brain, muscle, intestine, liver, adipose tissue and skin. The samples came from nine captive-bred wheatears collected at three different stages of the endogenous autumn migratory period: (1) lean birds prior the onset of migration, (2) during the fattening stage and (3) individuals at their migratory body mass plateau, when they have almost doubled their lean body mass. The sample structure used to build up the transcriptome of the Northern Wheatears concerning tissue composition and time guarantees the future survey of the regulatory genes involved in the development of the migratory phenotype. Through the pre-migratory period, birds accomplish outstanding physical and behavioural changes that involve all organ systems. Nevertheless, the molecular mechanisms through which birds synchronize and control hyperphagia, fattening, restlessness increase, immunity boosting and tuning the muscles for such endurance flight are still largely unknown. The use of RNA-Seq has emerged as a powerful tool to analyse complex traits on a broad scale, and we believe it can help to characterize the migratory phenotype of wheatears at an unprecedented level. The primary challenge to conduct quantitative transcriptomic studies in non-model species is the availability of a reference transcriptome, which we have constructed and described in this paper. The cDNA was sequenced by pyrosequencing using the Genome Sequencer Roche GS FLX System; with single paired-end reads of about 400 bp. We estimate the total number of genes at 15,640, of which  67% could be annotated using Turkey and Zebra Finch genomes, or protein sequence information from SwissProt and NCBI databases. With our study, we have made a first step towards understanding the migratory phenotype regarding gene expression of a species that has become a model to study birds long-distance migrations.

## Introduction

Each year billions of birds move twice a year between nesting areas and non-breeding regions. Some birds even cover distances of up to 15,000 km one way each season (Bairlein et al., 2012). To cope with the vast distances between breeding and non-breeding areas, birds typically engage in long endurance flights, which impose extraordinary morphological, physiological and behavioural challenges for the animals (Bicudo et al., 2010; Bairlein, Eikenaar & Schmaljohann, 2015).

The development of the “migratory phenotype” involves almost all organ systems (Piersma et al., 2005). Increased migratory restlessness, hyperphagia, fattening, the switch in their circadian rhythm and the ability to navigate over long distances are common characteristics of the migratory birds (Bairlein & Gwinner, 1994; Berthold, 1996; Gwinner, 1996). Such a complex phenomenon needs a holistic approach for its comprehension; which became possible only recently, with the availability of Next Generation DNA Sequencing techniques (hereafter NGS). A small number of genes which appear to be associated with migration have already been identified by transcriptome and genome analyses of several migratory birds; e.g., Willow Warbler *Phylloscopus trochilus*—a study that showed genes associated with neuronal signalling and calcium flux were regulated (Boss et al., 2016). In the same species, Lundberg et al. (2017) found genetic variants in the chromosomes 1 and 5 that match perfectly with the geographic distribution of populations with different migratory directions. In these genomic regions can be found cluster of genes related to fatty acid synthesis, a key metabolic process limiting of physical performance. Another research about subspecies of Swainson’s Thrush, *Catharus ustulatus* with different migratory behaviour, found gene islands probably related to speciation (Ruegg et al., 2014). Nevertheless, other works have failed in the search for specific genetic signals related to migration using candidate genes among multiple species (Lugo Ramos, Delmore & Liedvogel, 2017). It seems like the study of the adaptations for long-distance migrations needs more careful experimental design based in functional studies.

Even though the phenomenology of avian long-distance migrations is well-described, still, deep molecular studies on that respect are missing. Some of these adaptations might have medical relevance (Totzke, Hübinger & Bairlein, 1998). Many “normal” phenotypic changes in birds like autumnal and spring fattening or glucose metabolism, would be life-threatening for mammals. The genetic characterization of these complex traits is necessary for a reliable interspecific comparison of metabolic and physiological processes. To disclose the pathways beneath the birds’ migratory phenotype may guide researches to better understand and treat human diseases like obesity, diabetes and metabolic syndrome.

### The Northern Wheatear as a model to study migration

The Northern Wheatear (*Oenanthe oenanthe*, Linnæus, 1758) breeds across most of the Holarctic with all the populations wintering in sub-Saharan Africa (Del Hoyo, Elliott & Sargatal, 2005; Bairlein et al., 2012).

*O. oenanthe* is a solitary, nocturnal long-distance songbird migrant which develops the migratory phenotype with consistency in captivity since it has mostly genetic basis (Maggini & Bairlein, 2010; Maggini, Bulte & Bairlein, 2017). At the same time, the birds can be studied relatively easy in the field, which makes the wheatear an excellent model to study the endogenous mechanism that commands their preparation for migration (Bairlein et al., 2013; Bairlein et al., 2015).

The assembly and annotation of a reference transcriptome for wheatear is the primary necessary step in identifying differentially expressed genes related to the migration phenotype in different organs. The goal of this article is to report the first transcriptome of the Northern Wheatear, based on RNA-Seq data. It was annotated *de novo*, using the Turkey and Zebra Finch genomes and Uniprot and NCBI databases. As a source of mRNA, we took samples from captive-bred birds in different stages of their endogenous migration cycle: (1) before the onset of migratory fuelling, (2) during fuelling, and (3) when fat stores reached their maximum, i.e., when they have nearly doubled their pre-migratory body mass (Maggini & Bairlein, 2010). The partial transcriptome was constructed by pooling mRNA from the brain, muscle, intestine, liver, adipose tissue and skin from three birds at each stage. The sample was sequenced by pyrosequencing using the Genome Sequencer Roche GS FLX System.

We believe that our data will be a useful tool in the way to dissect the molecular basis of one of the most intriguing phenomenon in nature, birds’ long-distance migration.

## Materials and Methods

### Sample collection

We sampled four female and five male F1-hybrid offspring of parental populations of *Oenanthe oenanthe* originating from Norway and Morocco; bred in outdoor aviaries in the natural breeding period on the grounds of the Institute of Avian Research (Wilhelmshaven, Germany) and taken indoors as soon as they were independent of their parents. From mid-August onwards until euthanization in early December, the period when fuelling and migratory activity occur (Maggini & Bairlein, 2010), the birds were kept in individual cages (size 50 × 40 × 40 cm) under controlled conditions at a light-dark cycle of 12 h light:12 h dark, 20  ± 1 °C room temperature. The birds were fed with a standardized insectivore diet (Bairlein, 1986) supplemented with a few mealworms and *ad libitum* fresh water. The body mass was recorded twice a week to the nearest 0.1 g, early in the morning before the birds were offered food. The birds were euthanized following Guglielmo, Haunerland & Williams (1998) in middle August, middle October and early December. Before exsanguination at the jugular vein, the animals were anesthetized with an intramuscular injection of ketamine hydrochloride (40 µl: 25 g). All procedures were in accordance with the German Animal Welfare Act and approved by the Zweckverband Veterinäramt JadeWeser; the number of the permitting authority is 42508-Te. The samples are stored at the Institute of Pharmacy and Molecular Biotechnology (IPMB), Heidelberg University, collection numbers IPMB 66613-66666.

### RNA purification

Organs were dissected from freshly killed birds and stored in liquid nitrogen until RNA isolation. For RNA purification, we used the “GeneMATRIX Universal RNA Purification Kit” (Roboklon GmbH, Berlin, Germany), and for a final “clean-up”, the “RNeasy Fibrous Tissue Mini Kit” (Qiagen GmbH, Hilden, Germany). We followed the protocols recommended by the suppliers; moreover, we did not load more than 12 mg of tissue per column, DNAse treatments lasted 1 h at 37 °C, and all washing steps were repeated three times. The general recommendations for RNA isolation by Sambrook & Russell (2012) were considered.

To check the absence of genomic DNA, a PCR targeting the mitochondrial gene cytochrome b was carried out. The amplifications were undertaken in a 30 µl reaction volume containing 1.5 mM MgCl_2_, 100 µM dNTPs, one unit of Taq DNA polymerase (Pharmacia Biotech, Munich, Germany), 4 µL from the RNA isolation and 5 pmol of each oligonucleotide (Wink et al., 2002), mta1: CCCCCTACCAACATCTCAGCATGATGAAACTTG and mtfr: CTAAGAAGGGTGGAGTCTTCAGTTTTTGGTTTACAAGAC. The PCR program consisted of three steps: (1) denaturation at 94 °C for 5 min; (2) 35 cycles, including denaturation at 94 °C for 25 s, annealing at 50 °C for 2 min and extension at 72 °C for 2 min, followed by (3) final extension at 72 °C for 10 min. PCR products were isolated and visualized by agarose gel electrophoresis.

The RNA concentration, cleanliness and integrity were evaluated using the standard spectrophotometric measurement and agarose gel electrophoresis.

### Transcriptome sequencing and annotation

With the aim of sequencing a partial transcriptome of the Northern Wheatear, 54 RNA isolations [∼713 ng each] were pooled. The samples were derived from nine wheatears, three per time-point (middle August, October, and December 2014) and six different organs each: brain, liver, intestine, muscle, skin and adipose tissue.

The cDNA library preparation, sequencing, and transcriptome assembly were conducted by GATC-Biotech AG (Konstanz, Germany) using 38.5 µg [350.4 ng/µl] of total high-quality RNA (28s/18s ratio ≥ 1.7; RIN ≥ 8; A260/A280 ≥ 1.8). From the total RNA sample, poly(A) + mRNA was isolated, and the first-strand cDNA synthesis was primed with random hexamer primers (N6). Then the “454 adapters” A and B were ligated to the 5′ and 3′ ends of the cDNA. The resulting cDNA (N0-cDNA) was finally amplified with PCR (number of cycles = 11) using a proof-reading polymerase.

Normalization was carried out by one cycle of denaturation and reassociation of the N0-cDNA, resulting in N1-cDNA. Reassociated ds-cDNA was separated from the remaining ss-cDNA (normalized cDNA) by passing the mixture over a hydroxylapatite column. After the chromatography, the sscDNA was amplified by PCR (number of cycles = 6) (Shcheglov et al., 2007). For sequencing, cDNA with a size range of 500–800 bp was selected from N1-cDNA using a preparative agarose gel electrophoresis.

The cDNA was sequenced using the “Genome Sequencer Roche GS FLX System” (GATC-Biotech AG, Konstanz, Germany), which produced reads of up to 400 bp length. The reference transcriptome was assembled using the ”Roche GS De Novo Assembler”: Newbler (version 2.8). In principle, the assembler starts with a read, looks at its sequence, and searches the reading space for another read that contains an overlapping sequence. The overlap is specified by its length and the number or percentage of matching bases (Margulies et al., 2005; Miller, Koren & Sutton, 2010; Schliesky et al., 2012).

### Data pre-processing

Newbler was run using default parameters for reads cleaning and transcriptome assembly. Reads that contained homopolymers (60% over the entire length of the read represented by one nucleotide) and low-quality sequences (quality scores <20) were discarded. The primers, adapters and ambiguous residues (Ns) from both sides of the sequence were trimmed and the read length was filtered at 20 bp.

Assembly parameters: minimum overlap length = 40 bp and minimum overlap identity = 90%. Maximum number of contigs in an isogroup, default = 500, maximum number of isotigs in an isogroup = 100, and maximum number of contigs in one isotig = 100. The assembly was not checked for chimeras. The parameters Large or Complex Genome, Heterozygotic Mode, or Expected Depth were not used.

If the assembly of the cDNA raw data indicated 2 or more possible high-quality assemblies of the same sequence, it was presumed that these differences are due to alternative splicing and the contigs were grouped in the same isogroup as isotigs.

Once the assembly had been carried out, the reads that survived the quality control steps were remapped to the reference transcriptome using bwa-0.7.12-mem (Li, 2013). The sam/bam files conversion was conducted using samtools-1.3.1 (Li et al., 2009) with default parameters.

### Assessment of the completeness of the Oenanthe oenanthe transcriptome assembly

The transcriptome was assessed using the Benchmarking Universal Single-Copy Orthologs, BUSCO, version 3.0.2 (Kriventseva et al., 2014; Simão et al., 2015). BUSCO sets are groups of orthologous near-universally-distributed single-copy genes in each species, selected from OrthoDB (http://www.orthodb.org; Zdobnov et al., 2016) across eukaryotes, fungi, arthropods, vertebrates, and metazoans. Their common presence means that any BUSCO gene can be expected to be found as a single-copy ortholog in any newly-sequenced genome or transcriptome from the appropriate phylogenetic clade. The ortholog genes are classified as “Complete”, “Fragmented”, “Duplicated” or “Missing” according to the “expected-score” and “protein length distribution” values (Simão et al., 2015). The approach was conducted using the lineage dataset: aves_odb9 (Creation date: 2016-02-13, number of species: 40, number of BUSCOs: 4,915) and metazoa_odb9 (Creation date: 2016-02-13, number of species: 65, number of BUSCOs: 978). The analysis was conducted using the following parameters: Blast *E*-value = 0.001, limit = 3 and run mode: transcriptome. Other standard parameters were considered to evaluate the quality of the assembly, such as the number of unique genes, N50, average transcript length, transcript length distribution and overlapping with the CDS used as reference for the annotation and number of annotated genes.

### Transcriptome annotation

The annotation of the transcriptome was conducted at the German Cancer Research Center (DKFZ, Heidelberg, Germany) using the published genomes from the Turkey (*Meleagris gallopavo*) and Zebra Finch (*Taeniopygia guttata*), Ensembl release_75, cDNA FASTA file, February 2014. The alignment was conducted at the nucleotide level using the *E*-value = 0.001 as a threshold, and no additional filter was used. The reference transcriptome (contigs/isotigs) was further annotated using BlastX version 223 (Altschul et al., 1997) against the Swissprot database from UniProt Knowledgebase, (release 2014_05; 545,388 entries). For the alignment, an *E*-value = 0.001 was set. The annotation was limited to those transcripts that showed a protein-to-protein identity higher than 50%.

We will refer to a “contig” and “isotig” as analogous to an individual transcript. Isogroups are clusters of isotigs that might be inferred as splicing variants. Some isogroups cluster transcripts with different annotation status (either like a “characterized gene”, “uncharacterized”, “characterized with biological function” or classified as a “novel gene”); we call them “ambiguous”.

The non-annotated genes from the first analyses in 2014 were re-annotated in March 2016 using the non-redundant proteins from NCBI database, http://www.ncbi.nlm.nih.gov (O’Leary Nuala et al., 2016). Then, 497 new isotigs could be annotated. The criteria used for the annotation were: protein-to-protein identity >50% and alignment length ≥80 bp. All annotations were combined, and the hits that matched a “known protein” with the highest percentage of identity or alignment score were preferred to name the isotigs. If there was no alternative matching to an “uncharacterized protein”, this label was kept.

The gene ontology terms from UniProt database, http://www.Uniprot.org (Bastian et al., 2015) were used to identify relevant biological functions in the sequence data. A list of the unique UniProt IDs from the annotation was loaded into the “Retrieve/ID mapping” webpage’s menu and the “Biological function” and the associated GO ids were harvested.

The R programming language platform, version 3.2.2, was used for basics statistical analysis and plotting (R Core Team, 2017).

## Results

### Sequencing outputs

A total of 1,358,490 sequence reads were obtained from the Genome Sequencer Roche GS FLX System, corresponding to 597,735,000 nucleotides. After filtering the data, 81.18% of the reads remained and 75.69% were assembled in contigs/isotigs (Table 1). Furthermore, ∼89% of the reads were successfully re-mapped to the reference transcriptome. The transcriptome assembly yielded 17,539 isogroups, clustering 21,982 contigs/isotigs with an average size of 1,622 bp and N50 value of 2,165 bp; the largest isotig is 14,979 bp long.

**Table 1:** *O. oenanthe* transcriptome assembly report from Newbler 2.8.

	amount	%
Total of reads	1,358,490	
Aligned reads	1,102,879	81.18%
Assembled reads	1,028,291	75.69%
Partial reads	74,000	5.45%
Singleton reads	139,611	10.28%
Repeat reads	68,301	5.03%
Outlier reads	18,759	1.38%
Too short reads	29,262	2.15%

### Transcriptome assembly and annotation

When the maximum number of isotigs in an isogroup (default 100) was exceeded, these transcripts were grouped independently and identified as “contigs”. 44 of these contigs were clustered in 18 isogroups; among them, 12 transcripts have a biological function, and 32 are novel genes.

**Figure 1:**
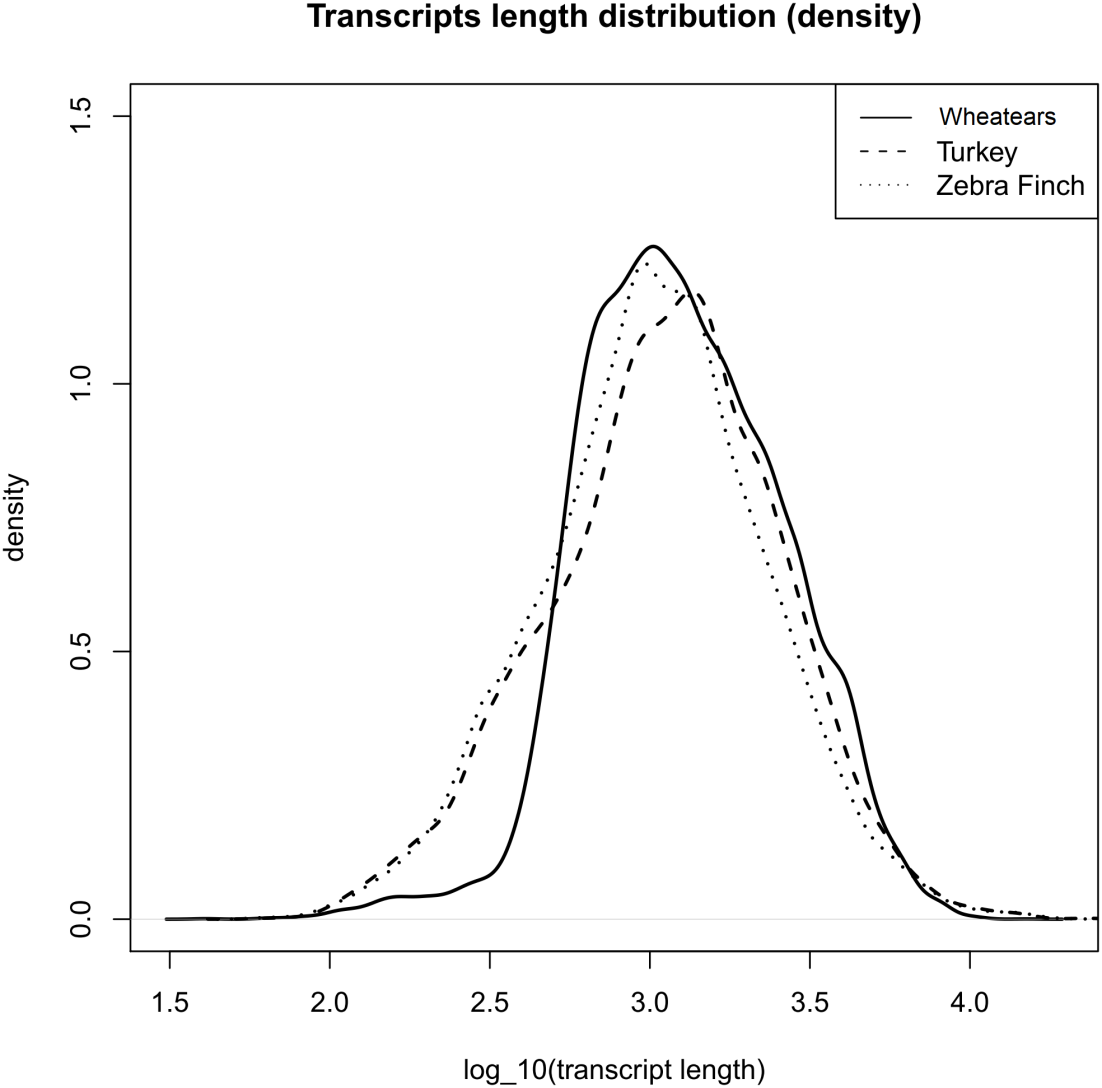
Transcript length distribution of *O. oenanthe*, *M. gallopavo* and *T. gutatta*, references used for the wheatear’s transcriptome annotation.

The mean length of the transcripts from *O. oenanthe* and the ones from the reference genomes, Turkey and Zebra Finch are similar, (Fig. 1).

Up to 396 isogroups are ambiguous, meaning they cluster isotigs with a different annotation status; we treat them separately to avoid confusion. Most of these isogroups (357) have a single protein name associated with non-annotated or uncharacterized isotigs.

For the rest of the isogroups (17,143), all the isotigs have a coherent annotation. Moreover, some isogroups could share the same Uniprot ID and different Uniprot IDs could share the same “gene name” as well. Approximately 67% of the total number of transcripts have orthologous genes in the protein and gene libraries used for the annotation and the residual 33% remain as non-annotated genes. The general information about the transcriptome annotation is summarized in Table 2 and the full annotation can be found in the supplements (Table S1). This Transcriptome Shotgun Assembly project (transcripts longer than 199 bp) has been deposited in the DDBJ/EMBL/GenBank database (http://www.ncbi.nlm.nih.gov/genbank), under the access code GFYT00000000. The version described in this paper is the first one, GFYT00000000. The raw data can be accessed in the “Sequence Read Archive” (SRA) database (http://www.ncbi.nlm.nih.gov/sra), BioProject: PRJNA408074.

**Table 2:** Summary of the *O. oenanthe* transcriptome annotation. Around 6.0% of the genes are “uncharacterized”, ∼49% have biological functions associated (GO) and ∼37% could not be annotated. The 396 ambiguous isogroups are not considered here.

**Transcriptome**	**Characterized**	**Char. with GO**	**Uncharacterized**	**Novel**	**Total**
Isogroups	1,442	8,355 (49%)	1,055	6,291 (37%)	17,143
Isotigs	1,757	10,449	1,198	7,016	20,420

The number of genes identified in *O. oenanthe* might not exceed 15,640, considering that all characterized isogroups with the same description belong to the same gene (7,898). Furthermore, it is assumed that all the novel (6,291), ambiguous (396) and un-characterized (1,055) isogroups are unique genes.

The smallest transcript found is the isotig 21,938 of unknown function (40 bp in length); the largest is the main apolipoprotein of chylomicrons and low-density lipoproteins: “Apolipoprotein B-100” (length 14,979 bp).

**Figure 2:**
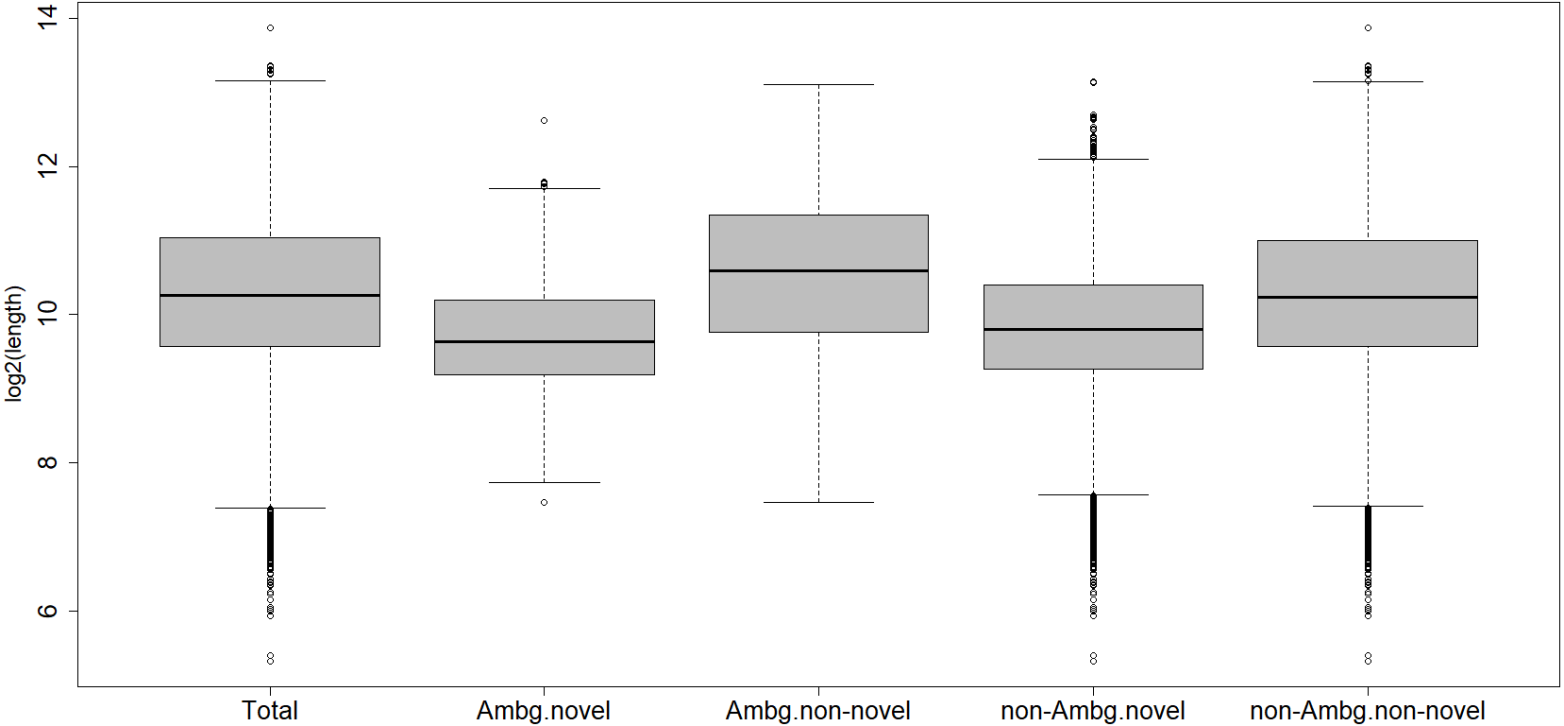
Length distribution of different subcategories of transcripts. Total, all transcripts; Ambg.novel, ambiguous and novel isogroups; Ambg.non-novel, ambiguous but annotated isogroups; non.Ambg.novel, novel unambiguous isogroups; non.Ambg.non-novel, annotated and unambiguous isogroups.

Within the ambiguous isogroups, the novel isotigs are significantly shorter than their annotated partners, with a mean length of 1,024 bp (Fig. 2), Student’s *t*-test *p*-value = 2.2 e^−16^. The characterized transcripts are, on average, 1,913 bp long, the ones with biological functions are a mean 1,924 bp long, the un-characterized 1,626 bp, and 1,621 bp is the mean for the whole transcriptome.

About 10,896 out of 12,206 characterized transcripts have a recognized putative biological function. Among the ambiguous isogroups, 746 out of 1,562 isotigs have an ontology term associated; all these 396 isogroups have at least one characterized isotig with a biological function.

Details regarding the validation of the assembly quality based on orthologous completeness are shown in Table 3.

**Table 3:** Orthologous completeness of the wheatear transcriptome.

	**Total BUSCOs**	**Complete BUSCOs**	**Complete and single-copy**	**Complete and duplicated**	**Fragmented**	**Missing**
Aves	4,915	2,364 (48.1%)	2,000 (40.7%)	364 (7.4%)	616 (12.5%)	1,935 (39.4%)
Metazoans	978	668 (68.3%)	606 (62.0%)	62 (6.3%)	107 (10.9%)	203 (20.8%)

**Figure 3:**
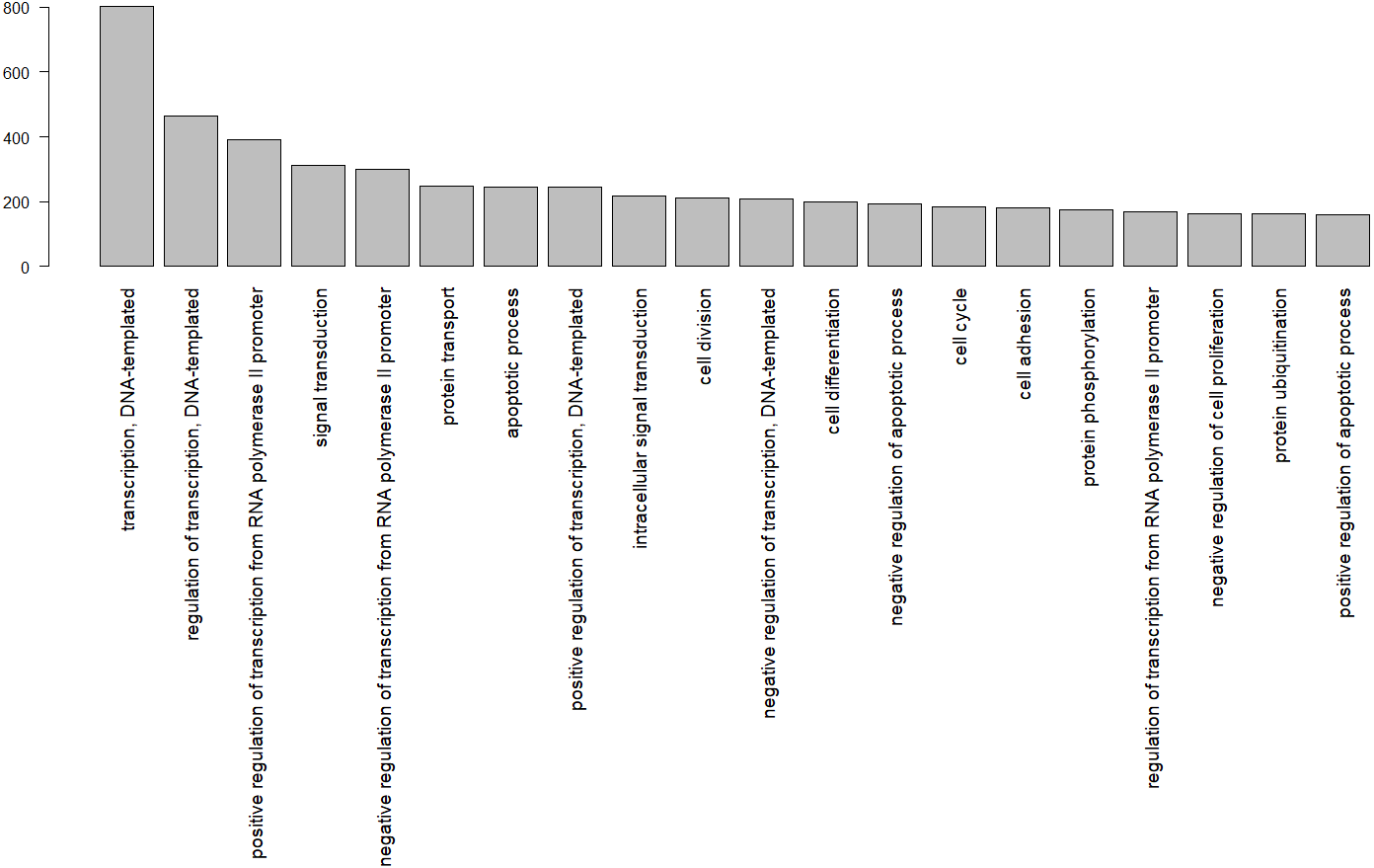
Gene ontology terms shared by at least 100 genes, identified by their Uniprot IDs in the wheatear transcriptome.

In total, 8,374 gene ontology terms were compiled (Table S2). Figure 3 shows the GO subcategories shared by more than 100 unique Uniprot.IDs.

## Discussion

The lack of a reference genome or transcriptome is one of the first challenges at the time to use NGS in studies involving non-model organisms. The assembly of the transcriptome of *O oenanthe*, by using the principal organs involved in, e.g., fattening (intestine, liver and adipose tissue), flight (muscle) and behavior (brain) along the pre-migratory season is not casual. It is the initial step for the understanding of how those processes are regulated at the level of gene expression. The transcriptome we are presenting here is partial, but we believe will be useful to illuminate the genetic architecture behind the development of the migratory phenotype.

The transcriptome of the Northern Wheatear was assembled *de novo*, creating isogroups with the closest related isotigs as putative splicing variants. Later, the transcripts were annotated individually; therefore, 396 isogroups cluster together annotated transcripts and novel ones; we called them ambiguous. Probably because these novel transcripts are smaller, the alignment outputs differ and consequently so do the results of the annotation (Fig. 2).

### Assessment of the quality of the transcriptome assembly

To build up a reliable transcriptome, *de novo*, for non-model organisms coming from natural populations is especially challenging. The indicators of quality should, ideally, yield information about accuracy, completeness, contiguity, chimerism and variant resolution. Honaas et al. (2016) have recommended a combination of metrics that best assess the quality of *de novo* transcriptome assemblies: the proportion of reads mapping to an assembly, the recovery of conserved, widely expressed genes, the N50 length, and the total number of unigenes. In practice, it is tough to find one assembly which excels in all aspects of quality (Bradnam et al., 2013; Wang & Gribskov, 2017).

The transcriptome of *O. oenanthe* was constructed using 75.7% of the original sequenced reads. Approximately 10% of the reads are singletons, and 1.4% are outliers. Analogous results were obtained using the same technology and a similar assembly strategy in other bird species (Peterson et al., 2012). These unassembled reads likely correspond to lowly-expressed transcripts with insufficient coverage to enable assembly; they are of low quality or aberrant reads. It has been found that, for assembling non-uniformly expressed transcripts, Newbler can report reads from highly expressed genes as singletons (O’Neil & Emrich, 2013), although the wheatears’ cDNA library was normalized previous sequencing. Around 89% of the reads that passed the quality control filters mapped back to the contigs/isotigs. We consider that number a good indicator of assembly quality since the final transcripts are the consensus sequences from non-genetically identical individuals, hybrids of two subspecies.

The transcripts have an average size of 1,621 bp and an N50 value of 2,165 bp. These parameters, taken as quality indicators, are controversial and should be used carefully when comparing different studies. They could change depending on the threshold used for transcript lengths and the propensity of the assemblers to create chimeras. N50 should reflect the real values of the mRNAs since, in *de novo* assemblies, it is unknown *a priori* (O’Neil & Emrich, 2013). The wheatears’ N50 and isotigs’ length values compare favourably with other *de novo* transcriptome assemblies (Singhal, 2013; Balakrishnan et al., 2013; Theissinger et al., 2016). Moreover, it lies between the value range of three sparrow transcriptomes: 1,942–4,072 bp (Balakrishnan et al., 2014). In that study, the differences in lengths were argued to be strongly influenced by the quality of the sample source, sequencing depth and reading types: single or paired-end. The last two aspects could be considered to improve our work in the future.

Our data was not filtered by sequence length, it will be a decision for future users as to which parameter thresholds are to be used for the analysis of the wheatears’ transcriptome. The shortest transcript with a biological function is a splicing regulator, the isotig 21,903: YTH domain-containing protein 1 (Xiao et al., 2016) with 106 bp and 90.91% of protein identity to its human orthologous gene. Just 35 transcripts are shorter than ytdc1 (40–105 bp), and all of them are classified as novel genes. We can not discard the presence of chimeras in our data; the most used tools to check for it require, for a reliable output, to have paired-end reads as primary data (Smith-Unna et al., 2016).

Incomplete transcript coverage can be reflected in the proportion of genes split into different isogroups, in our data approximately 19% (80% of them with just two isogroups). As an example, titin (TTN) the larger gene found in vertebrates (Opitz et al., 2003) is not the largest found in the wheatears’ transcriptome. Instead, TTN has 20 isogroups associated, most likely due to the lack of reads in some portion of the mRNA, preventing its full reconstruction.

### Completeness of the transcriptome of *O. oenanthe*

The comparison of *O. oenanthe* transcripts with the bird’s sets of Benchmarking Universal Single- Copy Orthologs, using BUSCO software, yielded ∼40% of missing genes. Just 2,000 “complete” orthologs out of 4,915 were identified in our dataset. A better recovery, approximately 70%, of common genes occurred when comparing our results with metazoans. Even though these results could be interpreted as a non-optimum sequencing coverage or assembly issues; there is no agreement about the extent to which well-conserved orthologous genes can serve as a proxy for the whole transcriptome (Peterson et al., 2012; Honaas et al., 2016). That the BUSCO sets of genes are based on information derived from genomes must be taken into account. We should not expect all the referenced genes to be found in the partial transcriptome of wheatears. Usually, the proportion of complete genes is higher when genome completeness is tested (Simão et al., 2015).

**Figure 4:**
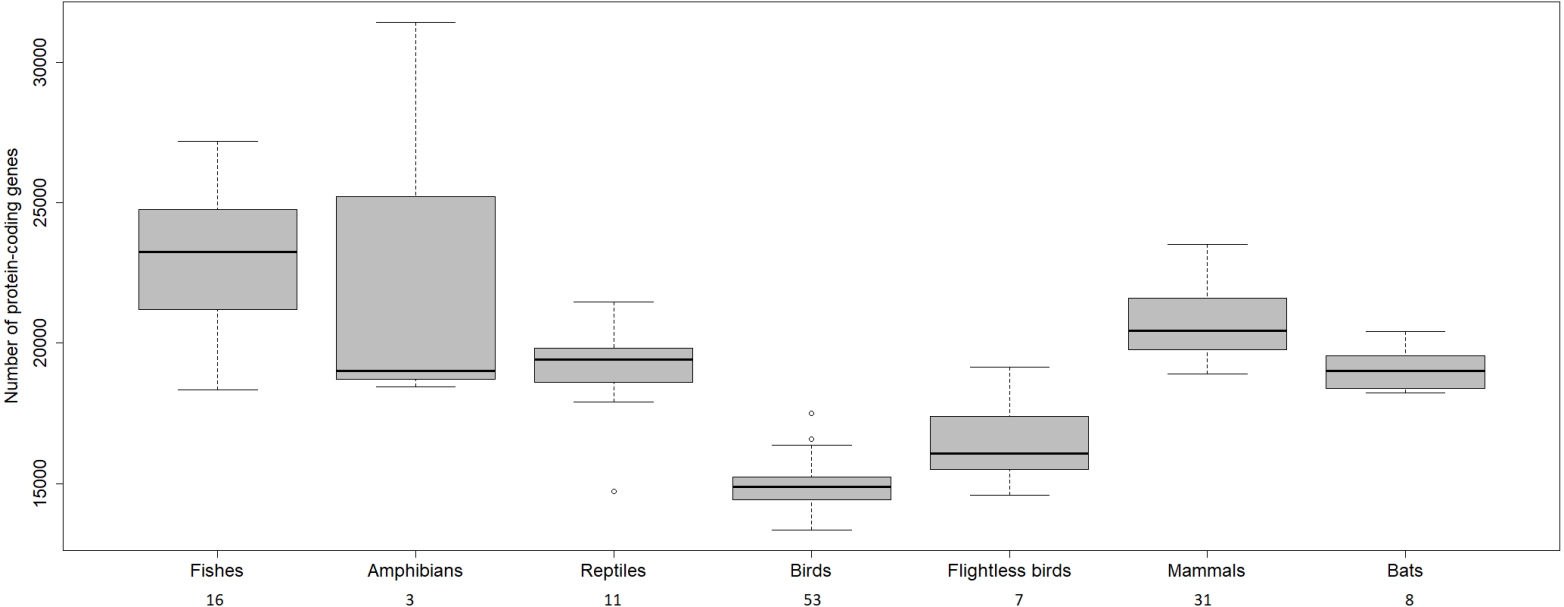
Number of protein-coding genes estimated for different vertebrates. The numbers below refer to the number of species used to construct the figure. Data source: www.ncbi.nlm.nih.gov/genome (O’Leary Nuala et al., 2016); www.ensembl.org/info/about/species.html ( Zerbino et al., 2018).

The transcriptome assembly of the wheatears yielded 21,982 contigs/isotigs, grouped into 17,539 isogroups. The putative number of coding genes identified in this communication, 15,640, is similar to other well-annotated birds’ transcriptomes, around 18,000 (see: Künstner et al., 2010; Balakrishnan et al., 2014; Finseth & Harrison, 2014; Koglin et al., 2017). We should mention that birds have suffered massive deletions of coding and non-coding regions in respect to reptilian sister clades and the rest of the vertebrates (Huang et al., 2013; Lovell et al., 2014; Zhang et al., 2014) (Fig. 4). We consider the finding of a reliable number of genes in wheatears a point in favour of the assembly usefulness. That is especially the case, as the main purpose of our immediate work will be the analysis of the regulated genes related to migration.

Among the biological functions associated with the annotated transcripts of *O. oenanthe,* there is a preponderance of terms related to transcription, signal transduction, and cell division. The analysis of the ontology of the genes related to migration in different tissues will be a topic for upcoming articles.

## Conclusions

We have established the first transcriptome of *O. oenanthe*, which is not complete but contains about 16,000 genes compared to ∼18,000 in other birds. More than 66% of the transcripts could be annotated. It will be an important base for further studies based on RNA-Seq of multiple organs and different migratory stages. The information coming from many other newly sequenced genomes and transcriptomes will help to further annotate this transcriptome.

##  Supplemental Information

10.7717/peerj.5860/supp-1 Table S1 Details of the annotation of the partial transcriptome of *Oenanthe oenanthe* The table includes the gene name (Description), Uniprot IDs, Isogroups, Frequency: number of isotigs/contigs associated to each isogroup, Contig/Isotigs IDs, Transcript length, Transcript sequence, Biological function and Classification according the annotation status: genes with biological function (ann.char.bf), genes annotated without biological function (ann.char), uncharacterized genes (ann.unchar), non-annotated genes (novel.gene). This Transcriptome Shotgun Assembly project (transcripts longer than 199 bp) has been deposited at DDBJ/EMBL/GenBank (www.ncbi.nlm.nih.gov/genbank) under the accession GFYT00000000; the version described in this paper is the first version, GFYT01000000. The raw data can be acceded in the Sequence Read Archive (SRA) database (www.ncbi.nlm.nih.gov/sra), BioProject: PRJNA408074.

10.7717/peerj.5860/supp-2 Table S2 Absolute frequency of contig/isotig associated to each ”Gene Ontology term” Absolute frequency of transcripts that share the same biological function. In total, 8,374 gene ontology terms, according to www.uniprot.org (2017), were compiled for the partial transcriptome of *Oenanthe oenanthe*.

## Additional Information and Declarations

### DNA Deposition

The following information was supplied regarding the deposition of DNA sequences: This Transcriptome Shotgun Assembly project in available at DDBJ/EMBL/GenBank under the accession GFYT00000000.

### Data Availability

The following information was supplied regarding data availability: The raw data have been provided as Supplemental Files.
